# Threshold response of mesophyll CO_2_ conductance to leaf hydraulics in highly transpiring hybrid poplar clones exposed to soil drying

**DOI:** 10.1093/jxb/ert436

**Published:** 2013-12-24

**Authors:** Guillaume Théroux-Rancourt, Gilbert Éthier, Steeve Pepin

**Affiliations:** ^1^Department of Plant Sciences, Horticultural Research Center, Université Laval, 2480 boul. Hochelaga, Quebec, QC, G1V 0A6, Canada; ^2^Department of Soil and Agri-Food Engineering, Horticultural Research Center, Université Laval, 2480 boul. Hochelaga, Quebec, QC, G1V 0A6, Canada

**Keywords:** Drought, internal CO2 conductance, leaf hydraulic conductance, Populus, stomatal limitation, water use efficiency.

## Abstract

Mesophyll conductance (*g*_m_) has been shown to impose significant limitations to net CO_2_ assimilation (*A*) in various species during water stress. Net CO_2_ assimilation is also limited by stomatal conductance to water (*g*_sw_), both having been shown to co-vary with leaf hydraulic conductance (*K*_leaf_). Lately, several studies have suggested a close functional link between *K*_leaf_, *g*_sw_, and *g*_m_. However, such relationships could only be circumstantial since a recent study has shown that the response of *g*_m_ to drought could merely be an artefactual consequence of a reduced intercellular CO_2_ mole fraction (*C*_i_). Experiments were conducted on 8-week-old hybrid poplar cuttings to determine the relationship between *K*_leaf_, *g*_sw_, and *g*_m_ in clones of contrasting drought tolerance. It was hypothesized that changes in *g*_sw_ and *K*_leaf_ in response to drought would not impact on *g*_m_ over most of its range. The results show that *K*_leaf_ decreased in concert with *g*_sw_ as drought proceeded, whereas *g*_m_ measured at a normalized *C*_i_ remained relatively constant up to a *g*_sw_ threshold of ~0.15mol m^–2^ s^–1^. This delayed *g*_m_ response prevented a substantial decline in *A* at the early stage of the drought, thereby enhancing water use efficiency. Reducing the stomatal limitation of droughted plants by diminishing the ambient CO_2_ concentration of the air did not modify *g*_m_ or *K*_leaf_. The relationship between gas exchange and leaf hydraulics was similar in both drought-tolerant and drought-sensitive clones despite their contrasting vulnerability to stem cavitation and stomatal response to soil drying. The results support the hypothesis of a partial hydraulic isolation of the mesophyll from the main transpiration pathway.

## Introduction

Attempts to establish significant relationships between physiological variables relating CO_2_ assimilation to water transport have been helpful for our understanding of leaf processes under different environmental conditions. For instance, Brodribb and Holbrook (2003, 2004) observed concerted decreases in net photosynthesis (*A*), stomatal conductance to water (*g*_sw_), and leaf hydraulic conductance (*K*_leaf_) in tropical tree species during periods of low soil water availability and/or high evaporative demand. These reductions in gas exchange have been considered a way to avoid major cavitation events by maintaining leaf water potential above a critical threshold (Sack and Holbrook, 2006), a mechanism also observed with stem hydraulic conductance (*K*_stem_) (Tyree and Sperry, 1988). Further, there is growing evidence of greater diurnal variation in *K*_leaf_ than in *K*_stem_, which suggests that *K*_leaf_ is more closely coupled to gas exchange (Brodribb and Holbrook, 2004; Sack and Holbrook 2006). Of the different leaf resistance components, xylem cavitation is the most important source of resistance to water transport in highly transpiring plants, whereas the resistance occurring outside the xylem represents about a third of the total resistance to leaf water movement (Sack *et al.*, 2004, 2005). Nonetheless, a decline in water transport outside the xylem generally occurs during drought stress due to a shift from the symplastic–transcellular to the apoplastic water pathways, which also produces a decrease in *K*_leaf_ and *g*_sw_ (Pou *et al.*, 2013). Thus, in highly vascularized plants such as hybrid poplar, it may be expected that during drought, leaf xylem cavitation would precede an eventual shift of water flow to the outer xylary water pathways. This in turn would lead to a decrease in *K*_leaf_ and gas exchange as a way to protect the leaf against excessive cavitation.

When considering how environmental conditions may affect CO_2_ transport inside leaves, one must take into account the mesophyll conductance (*g*_m_) pertaining to the CO_2_ diffusion path from the substomatal cavities to the chloroplast stroma (Ethier and Livingston, 2004; Evans *et al.*, 2009; Tholen *et al.*, 2012). Although an apparent dynamic regulation of *g*_m_ in changing microclimates or growth conditions has frequently been reported in recent years (reviewed in Flexas *et al.*, 2008), the physiological basis of such *g*_m_ responses and how these changes orchestrate themselves with parallel changes in *K*_leaf_ and *g*_sw_ remain poorly understood. For example, *g*_m_ and *g*_sw_ appear to decline in concert as a result of drought stress (Warren, 2008; Centritto *et al.*, 2009; Barbour *et al.*, 2010). However, some studies dealing mainly with short- to mid-term drought stress show little to no relationship between *g*_m_ and *g*_sw_ (Galle *et al.*, 2009; Resco *et al.*, 2009), thereby suggesting a possible threshold drought response of *g*_m_ in some species (see, for example, Duan *et al.*, 2011).

Because *K*_leaf_ is a measure of the ease with which liquid water perfuses through the leaf (Sack and Holbrook, 2006), and since part of this pathway (the mesophyll apoplastic–symplastic–transcellular pathway) is shared with CO_2_ diffusing towards the chloroplast as bicarbonate ion and dissolved gas (Kaldenhoff *et al.*, 2008), one would expect a functional linkage between *g*_m_ and *K*_leaf_, be it in the form of the hydration status of the cell, or even perhaps through convergence of aquaporin-mediated membrane transport (Maurel *et al.*, 2008; Uehlein *et al.*, 2012). Aasamaa *et al.* (2005) showed that *K*_leaf_ increases with mesophyll density, a trait known to influence *g*_m_ (Loreto *et al.*, 1992; Fleck *et al.*, 2010). Moreover, they suggested that membrane-associated traits such as membrane permeability, or simply the frequency of plasmalemmas and tonoplasts in the hydraulic flow path of the mesophyll, were most influential on *K*_leaf_. The possibility that membranes also exert strong control over *g*_m_ is currently a much debated subject (reviewed in Kaldenhoff, 2012). Recently, Ferrio *et al.* (2012) suggested that the relationship between *g*_m_ and *K*_leaf_ would only be noticeable below a certain threshold. Presumably, the decrease of *K*_leaf_ and *g*_m_ as a result of water stress would be due to an eventual reduction of plasma membrane H_2_O/CO_2_ permeability, which in turn would divert symplastic water transport through the more tortuous apoplastic pathway (see also Ferrio *et al.*, 2009).

Despite the plausibility of the hypothesis of Ferrio *et al*., recent modelling results by Tholen *et al.* (2012) offer perhaps a simpler alternative explanation. Indeed, they have theoretically demonstrated that a threshold-like decrease of *g*_m_ with respect to *g*_sw_ in response to drought or salinity stress is expected merely from the relative increase in photorespiratory CO_2_ release ensuing from the eventual reduction of the intercellular CO_2_ mole fraction (*C*_i_) at low enough *g*_sw_. Thus, the apparent threshold-like response of *g*_m_ to drought could merely be an artefactual consequence of a reduction of *C*_i_—not a reduction of, say, cell membrane permeability as Ferrio *et al.* (2012) suggested.

To examine possible functional links between *K*_leaf_, *g*_sw_, and *g*_m_, as well as to verify the possible occurrence of a threshold-like response of *g*_m_ with respect to the other two leaf variables, the time course of leaf gas exchange was determined in parallel to *K*_leaf_ and *g*_m_ in hybrid poplars of contrasting drought tolerance during a short-term drought. It was also determined whether a reduction of stomatal limitation via a low CO_2_ treatment affects the short-term response of *g*_m_ after 1 week of reduced irrigation. To avoid any artefactual CO_2_ effects on *g*_m_ during measurements, care was taken to conduct the latter under constant *C*_i_. It was hypothesized that *g*_m_, thus evaluated at a normalized *C*_i_, would remain relatively constant and not display a threshold-like response in relation to *g*_sw_ and *K*_leaf_, and that low CO_2_ treatment at the end of the drought period would increase *g*_sw_ in the short term, but not *g*_m_ and possibly not *K*_leaf_.

## Materials and methods

### Plant material and growing conditions

Frozen cuttings of Assiniboine [(*Populus*×’Walker’:*Populus deltoides* L.×*P.*×*petrowskiana* R.I. Schrod. ex Regel)×male parent unknown] and Okanese [(*P.*×’Walker’)*×P.*×*petrowskiana*], with low and high putative drought tolerance, respectively (Silim *et al.*, 2009), were thawed in water for 24h and sprouted in a mist chamber for ~3 weeks in a 2:1 perlite:Pro-mix growing medium (Premier Horticulture, Rivière-du-Loup, QC, Canada). Plants were transferred to a 2:1 coarse sand:Pro-mix soil in 7 l pots and moved to a greenhouse for ~3 weeks (23/21 °C day/night temperature, 55% relative humidity, 16h photoperiod). When plants reached ~60cm in height, they were transferred to a growth cabinet and acclimated for at least 7 d [22/17 °C day/night temperature, 60% relative humidity, 16h photoperiod, 800 μmol m^–2^ s^–1^ photosynthetic photon flux density (PPFD)]. A 20–20–20 complete nutrient solution was applied twice a week for a total of 200 ppm N per week per plant. Experiments described later were carried out in the same growth cabinet under the same growing conditions.

### Gas exchange and fluorescence measurement

Gas exchange measurements were carried out when plants had reached a leaf plastochron index (LPI) of at least 12 (Larson and Isebrands, 1971). Plants were measured between 9:00h and 12:00h with a LI-6400XT equipped with a 6400–40 Leaf Chamber Fluorometer (LCF, LI-COR Biosciences, Lincoln, NE, USA). Leaves were initially acclimated under 800 μmol m^–2^ s^–1^ PPFD, 25.0±0.2 °C leaf temperature, 380 μmol mol^–1^ leaf chamber air CO_2_ (*C*_a_), 17.5±0.5 mmol mol^–1^ water vapour mole fraction, and 250 μmol s^–1^ air flow rate. Net CO_2_ assimilation rate at ambient conditions (*A*_Ca=380_), stomatal conductance to water vapour (*g*_sw_), and intercellular CO_2_ mole fraction (*C*_i_) were recorded once gas exchange variables reached steady state. Photochemical efficiency of photosystem II (Φ_PSII_) was estimated by recording steady-state fluorescence (*F*_s_) and maximal fluorescence under a saturating flash (*F*_m_′) of ~10 000 μmol m^–2^ s^–1^ using:

$$\Phi_{\text{PSII}}=\left(F_{\text{m}}\prime –F_{\text{s}}\right)/F_{\text{m}}\prime$$ (1)

### Mesophyll conductance measurements and calibration

The variable J method (Harley *et al.*, 1992) was used to estimate *g*_m_ using:

$$g_{\text{m}}=A/\left\{C_{\text{i}}–\Gamma *\times \;\left[J_{\text{f}}+\text{8 }\left(A+R_{\text{d}}\right)\right]/\left[J_{\text{f}}–\text{4}\times \;\left(A+R_{\text{d}}\right)\right]\right\}$$ (2)

where *J*_f_ is the photochemical electron transport rate estimated from chlorophyll fluorescence, Γ* is the chloroplastic CO_2_ photocompensation point, and *R*_d_ is the non-photorespiratory mitochondrial respiration in the light, which was estimated from the intersection point of two detailed *A*–*C*_i_ curves performed at 500 and 150 μmol m^–2^ s^–1^ PPFD. For these ‘Laisk method’ measurements of *R*_d_, a larger 2×3cm leaf chamber and an air flow rate of 500 μmol s^–1^ were used to minimize chamber leaks (LI-COR, 2012). Since the intersection point of the *A*–*C*_i_ curves of the Laisk method can only give an apparent intercellular CO_2_ photocompensation point (*C*_i_*), this value is usually converted to its chloroplastic Γ* equivalent assuming (von Caemmerer *et al.*, 1994; Ethier and Livingston, 2004):

$$\Gamma *=C_{\text{i}}*+R_{\text{d}}/g_{\text{m}}$$ (3)

However, this relationship assumes that the great majority of the gross intracellular CO_2_ flux crossing the chloroplast envelope also experiences the diffusional resistance due to the cell wall and plasmalemma (*r*_wp_). As explained in detail in Tholen *et al.* (2012), this is very unlikely, especially around Γ* where the net outgoing flux *R*_d_ crossing the wall/plasmalemma is typically only ~20% of the intracellular flux entering the chloroplast. Under such conditions, Equation 3 may be re-written as:

$$\Gamma *=C_{\text{i}}*+R_{\text{d}}\left(r_{\text{wp}}–r_{\text{ch}}/0.\text{2}\right)$$ (4)

where *r*_ch_ is the diffusional resistance due to the chloroplast envelope and stroma. Given that current estimates put *r*_ch_ ≥ *r*_wp_ in non-sclerophylls (Uehlein *et al.*, 2008; Tholen and Zhu, 2011; Tholen *et al.*, 2012), it is clear that *C*_i_* is probably greater than Γ*, rather than smaller as Equation 3 has it (see Tholen *et al.*, 2012 for further discussion). Given such uncertainties, it was chosen here to estimate Γ* in hybrid poplar from previous *in vitro* determinations of the specificity factor (*S*_c/o_) of Rubisco purified from other woody deciduous species (*S*_c/o_=100–104; see Balaguer *et al.*, 1996; Bota *et al.*, 2002), which on average corresponded to a Γ* of ~38 μmol mol^–1^ at 21% O_2_. This Γ* value appears in accordance with Equation 4 as it is below the average *C*_i_* value found here for the poplar clones (40.3 μmol mol^–1^); it also compares very well with the popular tobacco Γ* values commonly used to model photosynthesis and estimate *g*_m_ (see von Caemmerer *et al.*, 1994; Bernacchi *et al.*, 2002). Furthermore, Galmès *et al*. (2006) have shown the unreliability of *in vivo* Γ* estimates under water stress conditions, whereas leaf respiration—in particular dark leaf respiration (*R*_n_)—appeared less affected by drought. Consequently, the Laisk method was only used to estimate *R*_d_ and establish a relationship with *R*_n_ under well-watered conditions, and then this relationship was used to estimate *R*_d_ from *R*_n_ measurements in all subsequent experiments (see next section).

**Fig. 1.**
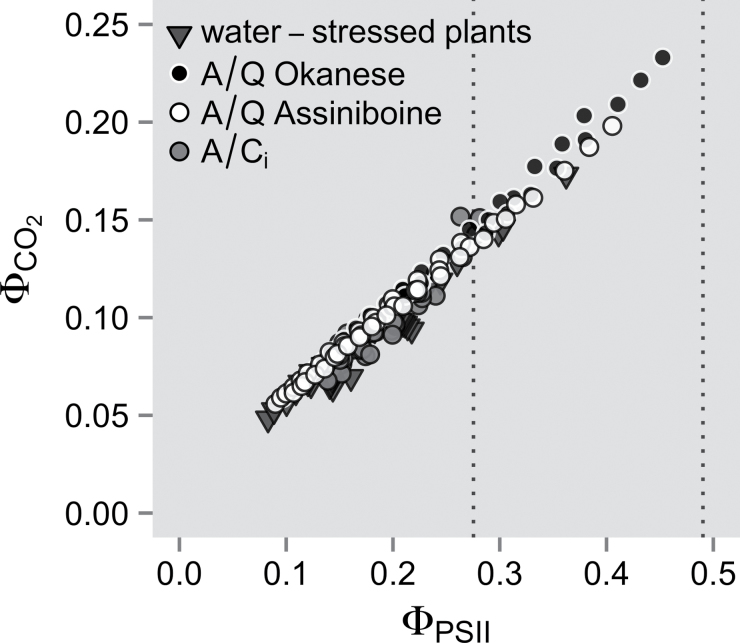
Relationship between the photochemical electron transport rate estimated from fluorescence measurements (*J*_f_) and gas exchange (*J*_CO2_) under 1% O_2_. Photosynthetic response curves to light (*A*/*Q*) and CO_2_ (*A*/*C*_i_) were carried out on three plants per clone, and the slope (*s*) and intercept of the relationship between Φ_CO2_ and Φ_PSII_ were used to calibrate *J*_f_ (see Equation 5). *A*/*C*_i_ data were pooled together for clarity. Water-stressed plants were also measured and were in a similar range to well-watered plants. The dotted lines indicate the range of Φ_PSII_ values observed during the short-term soil drying experiments (see Fig. 3). *A*/*Q* Okanese, Φ_CO2_=0.012+0.480×Φ_PSII_; *A*/*Q* Assiniboine, Φ_CO2_=0.016+0.450×Φ_PSII_; *A*/*C*_i_, Φ_CO2_=0.003+0.496×Φ_PSII_; water-stressed plants, Φ_CO2_=0.012+0.433×Φ_PSII_.

Calibrations of the relationship between the photochemical electron transport rate estimated from fluorescence measurements (*J*_f_) and gas exchange (*J*_CO2_) were carried out as described by Hassiotou *et al.* (2009). Briefly, CO_2_ response curves were carried out under non-photorespiratory conditions (1% O_2_) at a PPFD of 1000 μmol m^–2^ s^–1^ by progressively increasing *C*_a_ from 300 μmol mol^–1^ up to saturating CO_2_ (between 800 and 1000 μmol mol^–1^), which were followed by photosynthetic light responses curves, decreasing PPFD from 1500 to 500 μmol m^–2^ s^–1^ (Fig. 1). This allowed the calculation of *J*_f_ as:

$$J_{\text{f}}=[(\Phi_{\text{PSII}}\times \;s)+c]\;\times \text{ PPFD}$$ (5)

where *s* is the slope and *c* the intercept of the relationship of Φ_PSII_ and Φ_CO2_ at 1% O_2_, Φ_CO2_ being calculated as:

$$\Phi_{\text{CO2}}=\text{4 }\times \;\left(A+R_{\text{d}}\right)/\text{PPFD}$$ (6)

Calibrations carried out on water-stressed plants resulted in similar regressions; hence, data from both well-watered and stressed plants were pooled in the final (Fig. 1).

Nevertheless, such calibration of *J*_f_ under non-photorespiratory conditions did not always produce acceptable *g*_m_ values (see Gilbert *et al.*, 2012 for a detailed discussion of this issue), as many were negative or too high, particularly when the plants were under water stress. Consequently, an alternative method, in the authors’ view more likely to produce more reliable estimates of *s* under 21% O_2_, was evaluated. It is based on the premise that at 21% O_2_, *g*_m_ estimates should in theory vary little over a range of *C*_i_ values falling around the RuBP-limited *A*–*C*_i_ curve region where Φ_PSII_ is found to be constant (Tholen and Zhu, 2011). It was confirmed that this theoretically based assumption held for poplar using *g*_m_ measurements obtained with the standard isotopic method of Evans *et al.* (1986) (GE and SP, unpublished; see also Supplementary Fig. S1 available at *JXB* online). Thus, for this alternative *J*_f_ calibration method, detailed *A*–*C*_i_ curve analysis around the aforementioned *C*_i_ region of interest combined with chlorophyll fluorescence measurements under 21% O_2_ was used. *s* and *g*_m_ were then fitted over the *C*_i_ range of interest using the RuBP-limited photosynthesis equation given in Ethier *et al.* (2006) (see their equation 3) in order to minimize the sum of squares of errors. This method hence solves *s* (and so *J*_f_) and *g*_m_ simultaneously using the measured *A*–*C*_i_, Φ_PSII_, Γ*, and *R*_d_ as inputs.

Net CO_2_ assimilation and leaf transpiration rates measured with the LI-6400XT over the aforementioned *C*_a_ range were corrected for leaks following the method of Flexas *et al.* (2007a). Briefly, this consisted of subtracting the apparent CO_2_ assimilation rate of a dead leaf (immersed in boiling water for 5min) estimated at the corresponding leaf chamber CO_2_ concentration and air flow rate, taking care to allow the leaf to become fully equilibrated with the surrounding water vapour concentration of the chamber (set to 17.5 mmol mol^–1^). For H_2_O leak corrections, the apparent transpiration rate of the empty leaf chamber recorded at 17.5±0.5 mmol mol^–1^ water vapour concentration and appropriate air flow rate was subtracted from measured leaf transpiration rates.

### Post-calibration estimation of *R*_d_ from dark respiration measurements

Because during the short-term drought experiments the water potential of each leaf used for gas exchange needed to be subsequently rapidly measured with a pressure chamber to determine leaf hydraulic conductance (see ‘Leaf hydraulic conductance’ below), it was necessary to use a rapid proxy method to estimate *R*_d_ immediately following the leaf water potential (Ψ_leaf_) determination. For this, the leaf petiole was placed in a water-filled beaker and recut under water before clamping the leaf to a LI-6400 equipped with a 2×3cm leaf chamber, then covering it with a dark cloth to measure its *R*_n_ continuously for ~20min. To convert from *R*_n_ to *R*_d_, the following relationship previously established on the plants used for *J*_f_ calibrations was used:

$$R_{\text{d}}=\;0.\text{4787}R_{\text{n}}\left(n=\text{12},R^{\text{2}}=0.\text{53}\right)$$ (7)

Dark respiration was measured at a leaf chamber *C*_a_ set equal to the room *C*_a_ to avoid CO_2_ leakage effects. It was established that pressurization of leaves prior to the determination of dark respiration rates across a range of Ψ_leaf_ (–0.4 to –1MPa) had no impact on *R*_n_ compared with that of unpressurized leaves (data not shown).

### Short-term drought

Gas exchange of three plants per clone was repeatedly measured over a 12 d period of soil drying, for a total of nine measuring days. Plants were first measured under well-watered conditions, and the irrigation was then reduced on the following days to achieve a wide range of *g*_sw_ values. Measurements were carried on the same newly mature leaf (LPI=5 on day 1) throughout the experiment. Soil water potential (Ψ_soil_) was monitored on each measurement day using custom-made tensiometers and a digital reader (Tensimeter, Soil Moisture Equipment, Santa Barbara, CA, USA).

In addition, to get a better picture of the time course of *A*, *g*_sw_, *g*_m_, and *K*_leaf_, continuous measurements of gas exchange and plant and soil water potentials were performed on one plant per clone during 5 d of soil drying. Gas exchange was monitored during daytime with a LI-6400XT equipped with a 2×3cm adaptor chamber for a PAM-2000 chlorophyll fluorometer probe (Heinz Walz GmbH, Effeltrich, Germany), set at a flash intensity above 6000 μmol m^–2^ s^–1^. Through Equation 7, *R*_d_ was assumed to follow the observed decreasing trend of *R*_n_ as drought proceeded, dropping by ~40–50% after 5 d (from an estimated average initial *R*_d_ of ~1.1 μmol m^–2^ s^–1^). Soil water potential was measured using one tensiometer connected to a datalogger (CR7, Campbell Scientific, Logan, UT, USA). A leaf psychrometer (L-51, Wescor, Logan, UT, USA) was attached on an adjacent leaf to the one used for gas exchange to measure Ψ_leaf_, while another was attached to an adjacent dark covered leaf to measure Ψ_stem_. Psychrometer chambers were lightly lined with petroleum jelly to establish a good contact with the leaves (Savage *et al.*, 1983; Campbell and McInnes, 1999). Destructive Ψ_leaf_ and Ψ_stem_ measurements using a pressure chamber were carried out during the time course to correct the psychrometer readings according to a relationship between spot scale (psychrometer) and leaf scale (pressure chamber) water potential measurements established *a priori* (Ψ_leaf_=1.39 Ψ_psychrometer_, *n*=96, *R* ^2^=0.78; Ψ_stem_=0.45 Ψ_psychrometer_, *n*=113, *R* ^2^=0.54). To maintain a good contact between the psychrometers and the leaves, the psychrometers were removed daily, inspected, and cleaned if necessary, before resetting them to the leaves. Furthermore, the dark cover for Ψ_stem_ was removed daily after the final gas exchange measurements, ~4–5h before lights off.

### Reduction of drought-induced stomatal limitation using low *C*_a_

Reducing air [CO_2_] to very low concentrations around drought- or salt-stressed leaves leads to the opening of stomata to levels that result in a significant reduction of diffusional limitations (Centritto *et al.*, 2003; Bunce, 2007). This technique was thus used on water-stressed leaves to decrease stomatal limitation and monitor changes in *A*, *g*_m_, and leaf hydraulics.

Prior to soil drying and subsequent low *C*_a_ treatment, gas exchange of one leaf (LPI 5–7) was measured under well-watered conditions. Every other day, gas exchange parameters of the same leaf were measured to monitor changes in *g*_sw_ and to adjust irrigation based on a *g*_sw_ reduction of ~66% from the well-watered conditions. Soil water content (15cm TDR probe and Cable tester 1502C, Tektronix, Beaverton, OR, USA) and Ψ_soil_ were monitored daily to adjust irrigation between gas exchange measurements. Well-watered plants were also monitored as comparison.

After 7 d of reduced irrigation, the low *C*_a_ treatment was initiated. Gas exchange was measured first under ambient *C*_a_. The plant was then inserted in an ~13 l cylindrical cuvette designed to control the CO_2_ and H_2_O mole fractions around the plant. Between four and seven leaves, including the previously measured leaf, could be inserted in this large plant cuvette, while the upper and lower parts of the plant (~2–4 leaves each) remained under ambient growth chamber atmosphere (see Supplementary Fig. S2 at *JXB* online for a full description of the cuvette). The target leaf was clamped with the LI-6400XT LCF chamber inside the 13 l plant cuvette and both were sealed. The large plant cuvette operated as an open system, with an inflow of ~12 l min^–1^. The plant cuvette CO_2_ mole fraction was decreased to ~75 μmol mol^–1^ and the H_2_O mole fraction was maintained at ~17 mmol mol^–1^ by circulating the cuvette air through a H_2_O scrubbing Drierite™ column in series with a CO_2_ scrubbing column. A LI-840 CO_2_/H_2_O Analyzer (LI-COR Biosciences, Lincoln, NE, USA) was used to monitor the plant cuvette gas concentrations. Leaf temperature was maintained at 25±0.2 °C in the leaf chamber. Air temperature in the plant cuvette was ~26 °C, ~1 °C higher than the ambient air temperature outside the cuvette.

After *g*_sw_ had peaked and stabilized, the CO_2_ mole fraction was increased in the leaf chamber to reach a *C*_i_ of ~275 μmol mol^–1^ (see chlorophyll fluorescence calibration in the Results section) and *C*_a_ in the large plant cuvette was increased in parallel to match the *C*_a_ established in the leaf chamber. Once leaf gas exchange had reached steady state, three consecutive measurements were taken, each at least 2min apart.

### Leaf hydraulic conductance

For both the short-term drought and the low *C*_a_ experiment, *K*_leaf_ was estimated using the evaporative flux method:

$$K_{\text{leaf}}=E/\left(\Psi_{\text{stem}}–\Psi_{\text{leaf}}\right)$$ (8)

where *E* is leaf transpiration measured using the LI-6400XT, and Ψ_stem_ (stem water potential; measured on a dark-adapted leaf) and Ψ_leaf_ are measured using a Scholander-type pressure chamber (Model 610, PMS instruments, Albany, OR, USA) or the leaf psychrometers.

### Vulnerability curves

Vulnerability curves were carried out using a low-pressure flow meter (LPFM; Sperry *et al.*, 1988). Shoots were cut from plants in the growth chamber, put in a plastic bag, and brought to the lab. After an equilibration period of at least 5min, water potential was measured on a leaf. Stems were then cut under water, and stem segments of ~7cm long were prepared based on previous work by Harvey and van den Driessche (1997). Initial stem hydraulic conductivity (*K*_i_) was determined under a pressure head of 4.7 kPa by collecting water flowing out of the segment into a 10ml pipette and measuring the increasing water column in the pipette with a pressure transducer connected to a datalogger (CR23X, Campbell Scientific). When steady state was reached, stem segments were flushed at a pressure of 120 kPa for 7min to remove emboli before measuring maximum hydraulic conductivity (*K*_max_). Both flushes and conductivity measurements were carried out using a KCl (0.1M) solution filtered at 0.2 μm (Cai and Tyree, 2010). To reach more negative Ψ_stem_ values, shoots were left to dry in the lab and stem segments were measured upon reaching a desired value. Percentage loss conductivity (PLC) was calculated as:

$$\text{PLC}=\text{1}00\times \;\left(K_{\text{max}}–K_{\text{i}}\right)/K_{\text{max}}$$ (9)

The vulnerability curves were constructed by plotting PLC against Ψ_stem_, and then fitting a Weibull function:

$$\text{PLC}/\text{1}00=\text{1}–\text{exp}\left[–{\left(–\Psi_{\text{stem}}/b\right)}^{c}\right]$$ (10)

where *b* and *c* are fitting constants. Fitting was carried out using the *nls* function in R 3.0.0 (R Core Team, 2013).

### Statistical analysis

Statistical analyses were carried out with R 3.0.0. Mixed models were used to determine the hierarchical structure (i.e. plants within a measurement cycle) using the *nlme* procedure (Pinheiro *et al.*, 2013). Initial rates of leaf gas exchange were compared between clones to examine clonal differences. For the low *C*_a_ experiment, final measurements were analysed using a two-level factorial design (clone×low *C*_a_ treatment).

## Results

### Calibration of the chlorophyll fluorescence method for estimating the photochemical electron transport rates

**Fig. 2.**
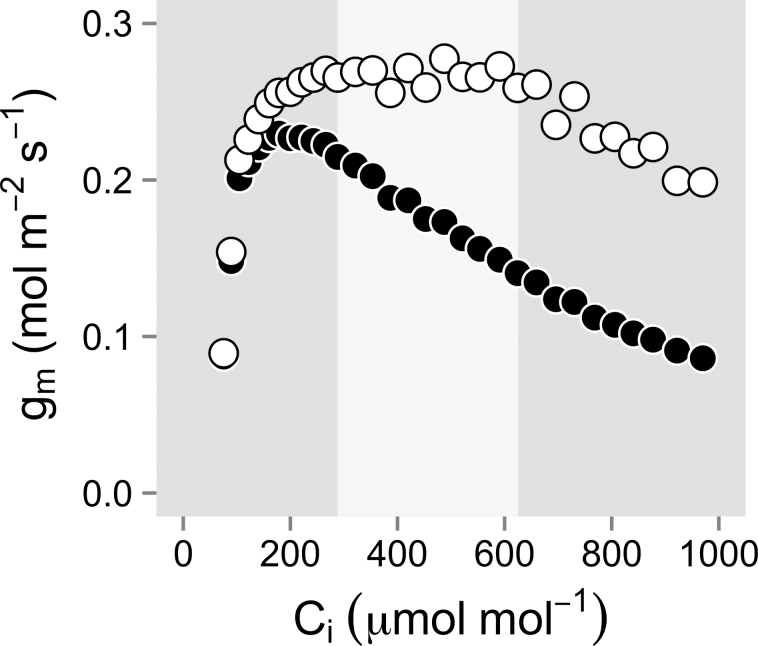
Apparent response of mesophyll conductance (*g*_m_) to intercellular CO_2_ mole fraction (*C*_i_) using the 1% O_2_ calibration method (filled circles) and the proposed 21% O_2_ *A*/*C*_i_ calibration method (open circles) on one Okanese leaf. The light coloured area represents the RuBP-limited range where Φ_PSII_ is found to be constant. Estimates of *s* under 1% and 21% O_2_ were 0.4799 and 0.4572, respectively.

Estimates of *g*_m_ based on the calibration of *J*_f_ under 1% O_2_ exhibited a positively skewed shape when plotted against *C*_i_, with a narrow maximum at ~200 μmol mol^–1^ (Fig. 2). However, using the proposed alternative calibration method at 21% O_2_, *g*_m_ estimates increased slightly and were more stable for several hundreds of ppm above a *C*_i_ of 200 μmol mol^–1^ (Fig. 2). Thus, in order to improve the estimates of *g*_m_ as *C*_i_ decreases under soil drying conditions, *C*_a_ was adjusted in the leaf chamber to reach a normalized *C*_i_ of 275 μmol mol^–1^ (hereafter named *C*_i_=275), a value consistently identified as the minimal *C*_i_ required to reach a stable and maximal *g*_m_. Using a normalized *C*_i_ value resulted in <5% of *g*_m_ estimates being discarded (i.e. negative or too high values) compared with 15% when measuring at ambient *C*_a_ (380 μmol mol^–1^). Overall, there was a slight difference in *s* values between clones (see Fig. 1) and the proposed calibration method yielded *s* estimates that were ~5% lower than those found under 1% O_2_ (Fig. 2).

### Gas exchange responses to short-term drought

**Fig. 3.**
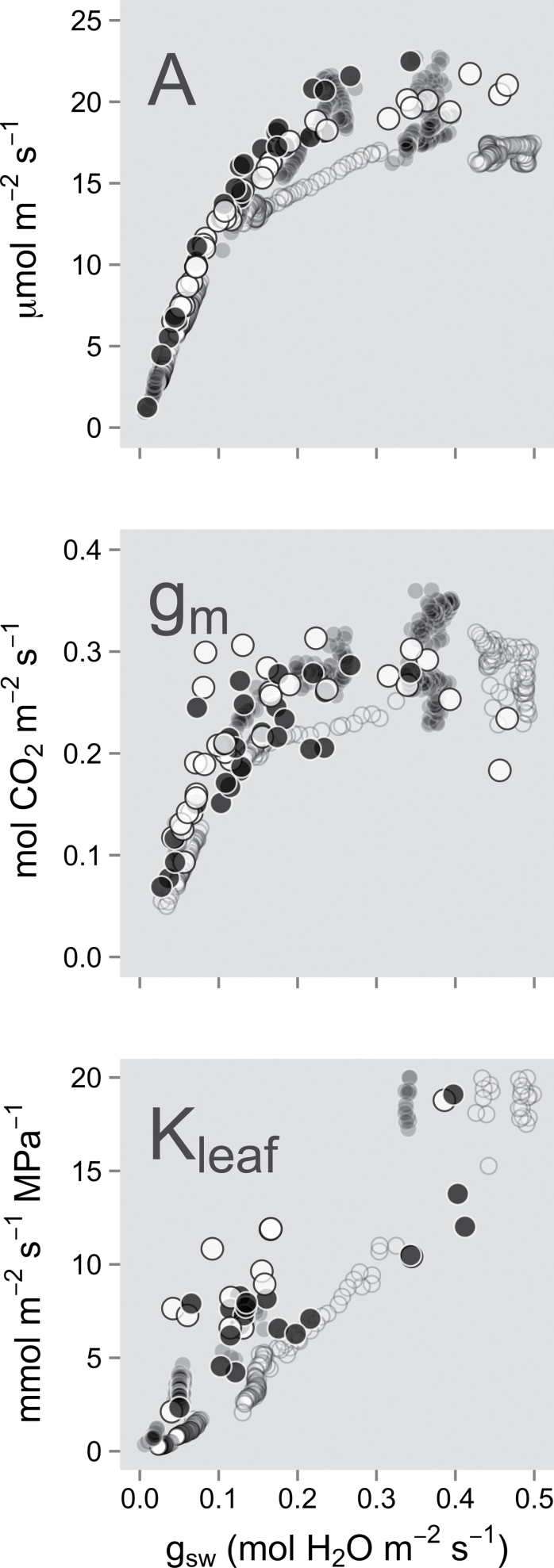
Changes in photosynthesis (*A*), mesophyll conductance (*g*_m_), and leaf hydraulic conductance (*K*_leaf_) in relation to stomatal conductance to water (*g*_sw_) during a short-term, moderate water stress. Three plants per clone were measured over 12 d of reduced irrigation, using the same leaf per plant. Assiniboine (drought sensitive; open circles), Okanese (drought tolerant; filled circles). Data from a separate 5 day soil drying experiment are also shown (translucent circles; one plant per clone; same colour code).

**Fig. 4.**
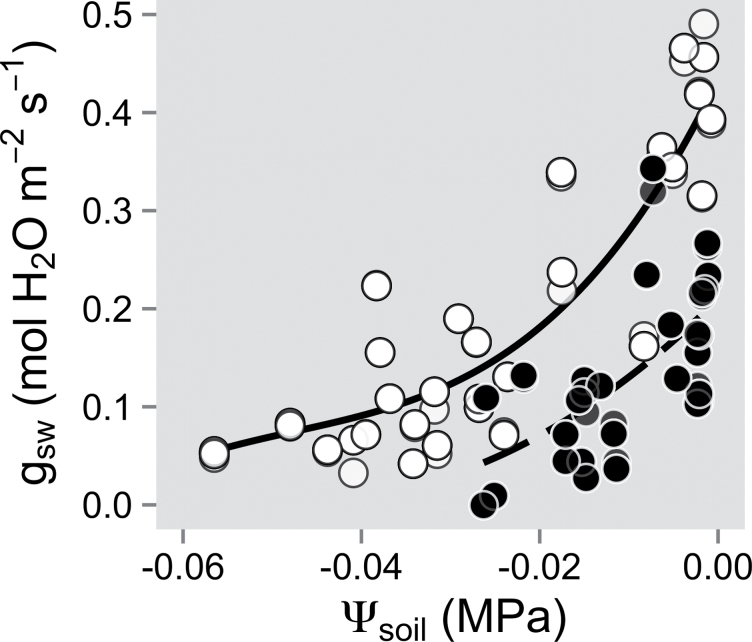
Responses of stomatal conductance (*g*_sw_) to decreasing soil water potential (Ψ_soil_) during the short-term drought experiment. A second-order polynomial curve was fitted for each clone [Assiniboine (open circles), *R* ^2^=0.79; and Okanese (filled circles), *R* ^2^=0.39; *P* < 0.0001].

Drought-sensitive Assiniboine and drought-tolerant Okanese had, under well-watered conditions, a similar photosynthesis rate under ambient *C*_a_ (*A*_Ca=380_; *F*_1,17_=2.11, *P*=0.16), and a similar normalized *g*_m_Ci=275_ (*F*_1,16_=2.93, *P*=0.11), although *g*_sw_ was slightly higher in Assiniboine (*F*_1,17_=3.62, *P*=0.07; Fig. 3). Twelve days of reduced irrigation gradually decreased photosynthesis (*A*_Ca=380_), *g*_sw_, transpiration (*E*), *K*_leaf_, and leaf and soil water potential (Ψ_leaf_ and Ψ_soil_) to very low values (e.g. a 90% decrease in *g*_sw_; see Figs 3 and 4). The decline in Ψ_leaf_, *E*, and *K*_leaf_ was concomitant with *g*_sw_, while the decline in *A* lagged behind *K*_leaf_. Normalized *g*_m_Ci=275_ remained constant over most of the drought-induced *g*_sw_ range, and decreased only when *g*_sw_ fell below a threshold of ~0.15mol m^–2^ s^–1^ (i.e. 68% and 56% of the maximum *g*_sw_ for Assiniboine and Okanese, respectively). However, because too low or near zero *g*_sw_ led to difficult if not impossible *g*_m_ fits, *g*_m_Ci=275_ values below 0.05mol m^–2^ s^–1^ were not included in the analysis as scatter increased tremendously below that threshold, even using the proposed calibration method.

Although the drought response of *A*_Ca=380_, *K*_leaf_, and *g*_m_Ci=275_ appeared similar for both clones when plotted against *g*_sw_, for any given Ψ_soil_, *g*_sw_ of Okanese was always lower than that of Assiniboine. Hence, for Okanese, the minimal *g*_sw_ was observed at a Ψ_soil_ of –0.03MPa, compared with –0.06MPa for Assiniboine (Fig. 4). Assiniboine reached higher *g*_sw_ values under well-watered conditions, but this did not translate into an increase in *A*_Ca=380_, and both clones exhibited a similar decline in *A*_Ca=380_ at common *g*_sw_. Similar gas exchange versus leaf hydraulic relationships to the 12 d reduced irrigation experiment were obtained over 5 d of water depletion during which *A*_Ca=380_, *g*_sw_, *K*_leaf_, and *g*_m_ (*C*_i_=225–275 μmol mol^–1^) were recorded continuously. Once again, *g*_sw_ and *K*_leaf_ decreased in concert, the decline in *A*_Ca=380_ lagged behind the two previous variables, and *g*_m_ remained constant up to a *g*_sw_ threshold of ~0.15mol m^–2^ s^–1^ (Fig. 3; see also Supplementary Fig. S3 at *JXB* online).

### Lowering *C*_a_ to decrease drought-induced stomatal limitation

**Fig. 5.**
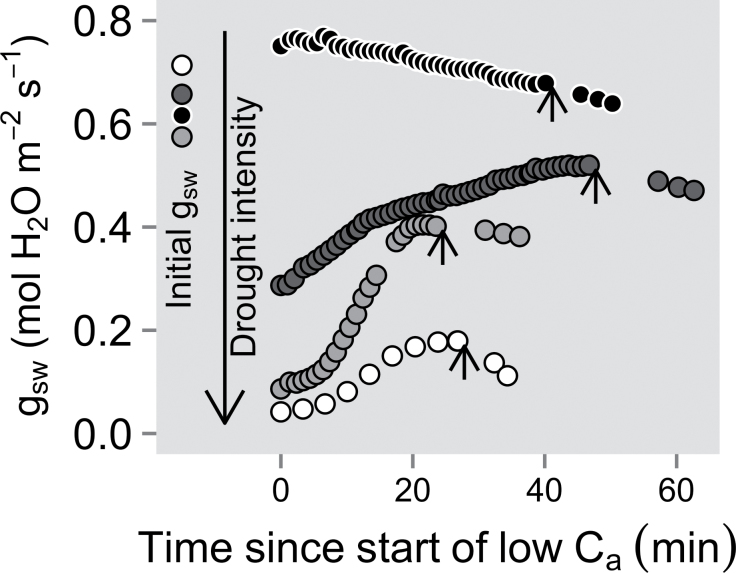
Time courses of stomatal conductance (*g*_sw_) when the upper foliage of well-watered and water-stressed Assiniboine plants was exposed to a reduction in ambient CO_2_ concentration (*C*_a_ ~75 μmol mol^–1^). Different levels of water stress were examined, expressed here as the percentage of *g*_sw_ relative to their initial well-watered state (*g*_sw_ini_) evaluated 7 d prior to the low *C*_a_ treatment (black circles, well-watered, where *g*_sw_ increased compared with *g*_sw_ini_; dark grey circles, 44% of *g*_sw_ini_; light grey circles, 15% of *g*_sw_ini_; and white circles, 6% of *g*_sw_ini_). The short arrows indicate when *C*_a_ was increased from ~75 μmol mol^–1^ to a CO_2_ concentration resulting in a *C*_i_ of ~275 μmol mol^–1^. The data points to the right of the arrows were measured at that *C*_i_, showing the high sensitivity of severely stressed plants to the return to near ambient *C*_a_.

After 7 d of reduced irrigation, *g*_sw_ was decreased by 75% on average in Assiniboine and 69% in Okanese. In both clones, lowering *C*_a_ around leaves resulted in a 3- to 4-fold increase in *g*_sw_ and *E*, which translated into a significantly more negative Ψ_leaf_ (Table 1). When *C*_a_ was reduced, *g*_sw_ reached a new steady state within 20–40min for water-stressed plants, and after 50–75min in well-watered plants (Fig. 5). Furthermore, *g*_sw_ rapidly declined after a return to a higher *C*_a_ in severely stressed plants (white dots in Fig. 5), while well-watered plants showed a more progressive return towards their initial *g*_sw_ level. In contrast to *g*_sw_ and Ψ_leaf_, no significant changes in *K*_leaf_, *A*_Ci=275_, and *g*_m_Ci=275_ were observed under low *C*_a_. There were no clonal differences in gas exchange responses to low CO_2_ treatment under reduced irrigation, although Assiniboine exhibited higher *K*_leaf_ than Okanese (Table 1).

**Table 1.** Effect of low air CO_2_ concentration around leaves on gas exchange and leaf hydraulics of water-stressed [foliar transpiration (E) <3 mmol m^–2^ s^–1^] hybrid poplar clones contrasting in drought tolerance

Clone (drought tolerance)	*C*_a_	*A*_Ci=275_ (μmol m^–2^ s^–1^)^*a*^	*g*_m_Ci=275_ (mol m^–2^ s^–1^)	*g*_sw_ (mol m^–2^ s^–1^)	*E* (mmol m^–2^ s^–1^)	*K*_leaf_ (mmol m^–2^ s^–1^ MPa^–1^)	Ψ_leaf_ (MPa)
Okanese	Ambient	18.7 (2.4)^*b*^	0.224 (0.042)	0.115 (0.053)	1.65 (0.63)	6.43 (2.1)	–0.87 (0.07)
(tolerant)	Low	20.9 (2.4)	0.270 (0.036)	0.325 (0.053)	4.08 (0.63)	9.28 (2.0)	–0.97 (0.07)
Assiniboine	Ambient	21.7 (2.3)	0.285 (0.037)	0.119 (0.051)	1.64 (0.59)	12.2 (2.2)	–0.76 (0.07)
(sensitive)	Low	15.0 (2.6)	0.204 (0.040)	0.206 (0.058)	3.07 (0.68)	14.5 (2.9)	–1.06 (0.08)
*P*-values^*c*^	Clone	0.69	0.96	0.22	0.34	0.04	0.66
	*C*_a_	0.35	0.59	0.007	0.007	0.21	0.01
	Clone×*C*_a_	0.06	0.10	0.23	0.44	0.90	0.21

^*a*^ Photosynthesis (*A*_Ci=275_) and mesophyll conductance (*g*_m_Ci=275_) measured at a normalized *C*_i_ of 275 μmol mol^–1^, stomatal conductance to water (*g*_sw_), transpiration (*E*), leaf hydraulic conductance (*K*_leaf_), and leaf water potential (Ψ_leaf_).

^*b*^ Standard error in parentheses.

^*c*^ Numerator d.f. = 1; denominator d.f. = 20 (*A*_Ci=275_, *g*_sw_, Ψ_leaf_, E), 17 (*g*_m_Ci=275_), 11 (*K*_leaf_).

### Vulnerability curves

**Fig. 6.**
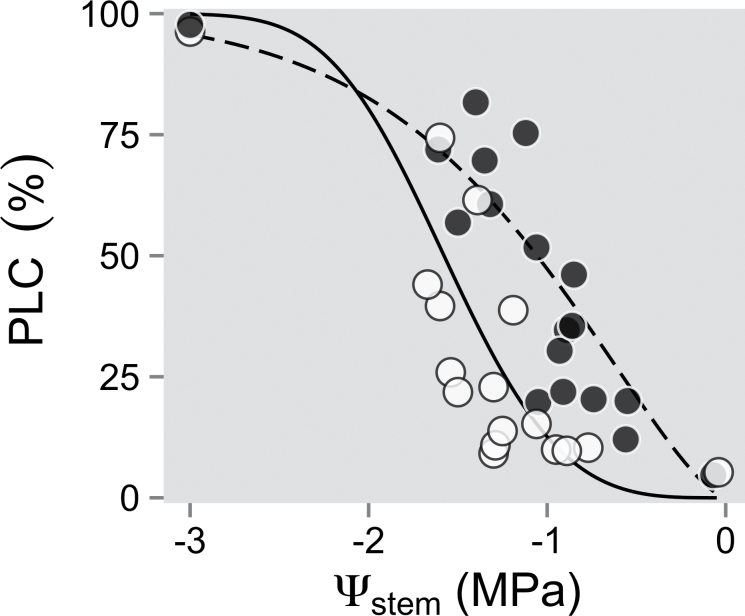
Stem vulnerability curve to cavitation (PLC: percentage loss conductivity) in drought-tolerant Okanese (filled circles) and drought-sensitive Assiniboine (open circles) hybrid poplar clones.

Since each clone exhibited a similar *A* versus *g*_sw_ response to drought, as well as a similar response to low *C*_a_ exposure, vulnerability to cavitation was measured to determine further the clonal differences in water stress response. Drought-tolerant Okanese exhibited a higher reduction in *K*_*s*tem_ over the observed Ψ_stem_ range compared with Assiniboine (Fig. 6). For instance, the PLC was up to 50% in Okanese upon reaching a Ψ_stem_ of –1MPa, while Assiniboine had ~15% PLC at the same potential. However, PLC increased rapidly after –1.2MPa Ψ_stem_ in Assiniboine to reach similar PLC values to Okanese near –1.5MPa (Fig. 6). Nevertheless, values of Ψ_leaf_ observed throughout the experiment were on average between –0.6MPa and –0.9MPa (first and third quartiles) for both clones, and Ψ_stem_ ranged between –0.3MPa and –0.6MPa. Thus, both clones usually did not reach a PLC of 50% but suffered only ~25% loss of conductivity under the moderate drought conditions of this study.

## Discussion

### Methodological considerations on the estimation of *g*_m_

Potential methodological errors and the underlying assumptions necessary in the estimation of *g*_m_ using the variable J method were discussed initially by Harley *et al.* (1992), and subsequently elaborated upon by several other authors (e.g. Warren 2008; Pons *et al.*, 2009; Gilbert *et al.*, 2012). Theoretically, positively skewed *g*_m_ versus *C*_i_ curves like those observed in this study when following the standard 1% O_2_ *J*_f_ calibration protocol (see also Flexas *et al.*, 2007b; Hassiotou *et al.*, 2009; Gilbert *et al.*, 2012) are predicted to result from small overestimations of *J*_f_, and/or possibly from underestimations of Γ*, or of *R*_d_ (see Harley *et al.*, 1992; Gilbert *et al.*, 2012). However, Tholen *et al.* (2012) demonstrated that even if all relevant parameters in the variable J method equation were exact, *g*_m_ would still be expected to decrease apparently sharply when the photorespiratory CO_2_ flux rises at low *C*_i_. One way to alleviate this artefactual CO_2_ sensitivity problem is by measuring *g*_m_ at a normalized *C*_i_, preferably around the *g*_m_ versus *C*_i_ curve region where *g*_m_ is expected to peak (i.e. the narrow *C*_i_ range where estimates of *g*_m_ are expected to be least affected by small errors of *J*_f_, etc.; see Fig. 2). However, even after taking this precaution, it was noted that ~5% of the initial *g*_m_ estimates for leaves subjected to water stress were deemed unreliable. One possibility that could explain this poor model performance is that the 1% O_2_ calibration method incompletely accounts for alternative electron sinks under 21% O_2_; especially under water stress (Makino *et al.*, 2002; Kitao *et al.*, 2003; Laisk *et al.*, 2006). In an attempt to address this possibility, we tested an alternative *J*_f_ calibration method performed under 21% O_2_, and which solves *s* and *g*_m_ concurrently from the CO_2_ response of photosynthesis and Φ_PSII_ in the RuBP-limited *A*–*C*_i_ curve region where photorespiration is expected to be low enough to exert little influence on *g*_m_ (see Fig. 2, and further details in the Materials and methods). From the present assessment, this alternative *J*_f_ calibration procedure yielded slightly lower photochemical electron transport rates than the 1% O_2_ calibration method, which resulted in ~20% higher *g*_m_ estimates, but did not improve the rejection of data under water stress conditions. Nevertheless, it is believed that calibrating Φ_CO2_ under 21% O_2_ should better account for potential alternative electron sinks under normal atmospheric conditions while greatly simplifying the calibration procedure.

**Fig. 7.**
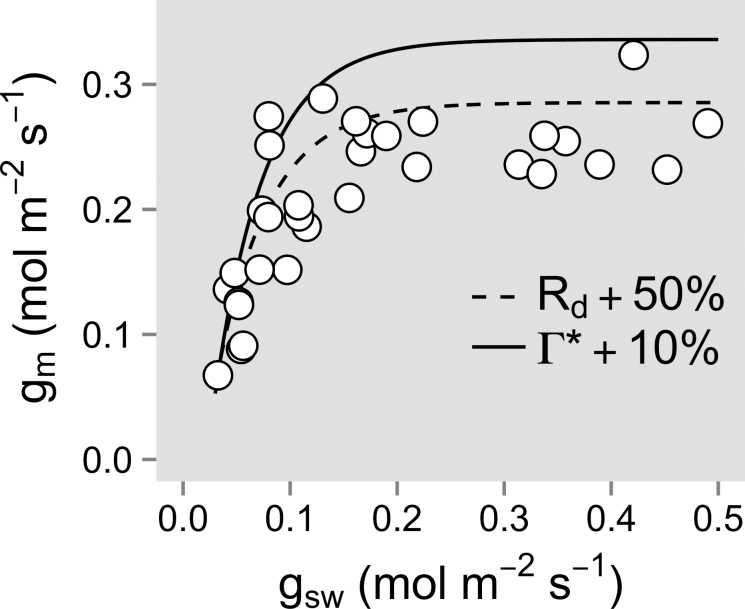
Sensitivity of mesophyll conductance (*g*_m_) estimates to a 10% increase in chloroplastic CO_2_ photocompensation point (Γ*, solid line) or a 50% increase in non-photorespiratory mitochondrial respiration in the light (*R*_d_, dashed line). Asymptotic fits of the simulated data were used to represent the observed variations. Data from Assiniboine in Fig. 3 were used (original data: open circles).

To evaluate possible *g*_m_ inaccuracies introduced by errors in the estimation of Γ* and *R*_d_, an analysis of the sensitivity of *g*_m_ to changes in Γ* (±10%) and *R*_d_ (±50%) was performed. As already pointed out by others (e.g. Harley *et al.*, 1992; Pons *et al.*, 2009), *g*_m_ was significantly more sensitive to potential errors in Γ* in comparison with *R*_d_ (see Fig.7)—in the present simulations, a 120% increase in *R*_d_ was necessary to obtain a *g*_m_ value corresponding to a +10% change in Γ*. However, because the estimation of *g*_m_ was restricted to a narrow *C*_i_ range neighbouring 275 μmol mol^–1^, such errors in Γ* and/or *R*_d_ are not expected to change the conclusions regarding the delayed threshold response of *g*_m_ with respect to *g*_sw_ (see Fig. 7).

### Threshold response of *g*_m_ to drought

Drought stress usually decreases *A*, *g*_sw_, and *K*_leaf_ concomitantly (Brodribb and Holbrook, 2006; Brodribb *et al.*, 2007; Warren, 2008; Galle *et al.*, 2009; Barbour *et al.*, 2010). Drought stress has also been shown to decrease *g*_m_ monotonically with respect to *A* and *g*_sw_ (Flexas *et al.*, 2007b, 2008; Warren, 2008; Perez-Martin *et al.*, 2009), although Flexas *et al.* (2002) reported a lower rate of *g*_m_ decrease at *g*_sw_ >0.15mol H_2_O m^–2^ s^–1^ in grapevine. However, these declines in *g*_m_ may only be circumstantial since a recent study has shown that *g*_m_ is expected to decrease apparently sharply in the absence of any change in intraleaf diffusional resistance component, once the photorespiratory rate of a water-stressed leaf rises significantly due to stomatal closure and ensuing reduction in *C*_i_ (Tholen *et al.*, 2012). Measuring *g*_m_ at a normalized *C*_i_ alleviates this effect, which allowed detection of a threshold response of *g*_m_ with respect to *g*_sw_ (~0.15mol H_2_O m^–2^ s^–1^), thereby confirming the observations of Ferrio *et al.* (2012). This recent study demonstrated a strong linear relationship between *g*_m_ and *K*_lamina_ (hydraulic conductance of the leaf lamina), but only when *K*_lamina_ fell below 8 mmol H_2_O m^–2^ s^–1^ MPa^–1^ (~70% of maximum value) as a result of water stress. Above this threshold point, corresponding to *g*_m_ values ≥0.2mol m^–2^ s^–1^, the relationship did not hold, as the effective water path length influencing *K*_lamina_ reached a constant baseline value. Hence, the *g*_m_–*K*_lamina_ sensitivity region corresponded to the point where the water path length between the xylem and the evaporative surface increased significantly above this baseline value.

The small difference in *g*_m_ threshold between the study of Ferrio *et al*. and the present study could arise from the different species and their respective hydraulic connectivity, but also because a constant *C*_i_ was used for *g*_m_ measurement with the variable J method (Harley *et al.*, 1992). Using a normalized *C*_i_ improved *g*_m_ estimates, as they reached similar values between clones. Nonetheless, in both studies, *g*_sw_ responded rapidly to early signs of water stress, and *g*_m_ followed afterwards. Concomitantly, Φ_PSII_, although not an ideal proxy for *g*_m_, has shown a delay in decrease as Ψ_leaf_ declined, beginning its descent often past a *g*_sw_ reduction of 50% or even 80% (Brodribb and Holbrook, 2003), as observed in the present study (Fig. 3). Hydraulic compartmentalization of the mesophyll might have contributed to this delayed response of *g*_m_. Two factors are important when considering compartmentalization: the linkage between the xylem and the epidermis, and the degree of uncoupling of the mesophyll cell to the hydraulic apparatus (Zwieniecki *et al.*, 2007). Thus, stomata, sensing early signs of water stress through small changes in Ψ_leaf_, could have started to close while more negative Ψ_leaf_ values (i.e. more severe stress) may have been needed to initiate a response in mesophyll cells or chloroplasts, leading to a decrease in *g*_m_. Zwieniecki *et al.* (2007) explained that such hydraulic compartmentalization would allow mesophyll cells to be buffered against short-term changes in leaf water status, a beneficial characteristic to C-assimilating cells. Also, the bulk of mesophyll cells have been shown to be rather unresponsive to exogeneously applied abscisic acid (ABA) (Shatil-Cohen *et al.*, 2011), which reinforces the idea that mesophyll cells are somewhat isolated from the main hydraulic path linking the xylem conduits and their bundle sheath extensions to the epidermis and stomata. Such structural considerations may act in conjunction with membrane-associated mechanisms such as gating of aquaporin channels and ensuing reduction of the symplastic water transport (Cochard *et al.*, 2007; Almeida-Rodriguez *et al.*, 2010; Pou *et al.*, 2013), which might eventually divert more water to the more tortuous apoplastic pathway (Ferrio *et al.*, 2012). The above model appears to fit well the hydraulic response of the hybrid poplar leaves despite the fact that it makes little mention of xylem cavitation. Certainly, the present results show that Ψ_stem_ did not reach low enough values to induce substantial stem cavitation (see also Secchi and Zwieniecki, 2010), but this does not necessarily mean that significant leaf cavitation could not have occurred. Indeed, Sack *et al.* (2004, 2005) reported that the xylem-related component of total leaf resistance dominates in highly transpiring plants such as hybrid poplar, and recently Johnson *et al.* (2012) demonstrated that in some species leaf xylem cavitation may account for most of the decrease in *K*_leaf_ under drought.

### Exposure to low *C*_a_ decouples *g*_s_ and *g*_m_

Centritto *et al.* (2003) proposed a method to separate diffusional limitations from non-diffusional limitations in salt-stressed plants: they lowered *C*_a_ to force stomata to open, thus reducing stomatal limitation upon returning to ambient *C*_a_. Surprisingly, the low *C*_a_ treatment appeared to reduce the mesophyll limitation just as rapidly as the stomatal limitation. However, the *C*_i_ values at which Centritto *et al.* (2003) evaluated *g*_m_ after pre-conditioning the stressed plants to low *C*_a_ was appreciably higher than before the low *C*_a_ treatment. Thus, because *g*_m_ was not evaluated at a normalized *C*_i_ in this study, it is likely that the conclusions of Centritto *et al.* (2003) about the rapid response of *g*_m_ to salt stress were confounded by the low *C*_i_ photorespiratory artefacts described in Tholen *et al.* (2012). In the present case, *C*_i_ was controlled close to 275 μmol mol^–1^ throughout and no significant change in *g*_m_ in response to low *C*_a_ pre-conditioning was detected.

Apart from its obvious consequences on gas exchange, low *C*_a_ treatment has major consequences for the leaf water status. As in the present study, Bunce (2007) reported a decrease (more negative) in Ψ_leaf_ using low *C*_a_ on a variety of water-stressed herbaceous and woody plants. Interestingly, the hybrid poplar plants used here reached a common Ψ_leaf_ value (approximately –0.92MPa to –1.01MPa) under low *C*_a_ regardless of drought intensity, which suggests that the degree of stomatal opening could have been modulated to keep Ψ_leaf_ above a catastrophic cavitation level (see also the results of Tardieu and Simonneau, 1998). If leaf xylem cavitation and concomitant decrease in Ψ_leaf_ were responsible for the decline in *K*_leaf_ under drought, *K*_leaf_ would have been expected to decrease more as a result of the low *C*_a_ treatment since Ψ_leaf_ was further reduced (see Table 1 compared with Supplementary Fig. S4 at *JXB* online). However, it did not, and similar observations by Bunce (2007) led him to question the dominant role of leaf cavitation in drought-induced *K*_leaf_ decline. One recent alternative explanation is a possible dual regulation of *g*_sw_ and *K*_leaf_ by ABA under soil drying conditions (Pantin *et al.*, 2013). Indeed, in the present study, water-stressed plants exhibited greater stomatal sensitivity to changes in *C*_a_ than well-watered plants (Fig. 5), a trait normally associated with the presence of higher levels of ABA (Raschke, 1975; Bunce, 2007).

Cochard *et al.* (2007) reported that aquaporin expression in the leaf vein bundle sheaths could be a major control point on leaf water status, observing a substantial drop in Ψ_leaf_ when aquaporin activity was inhibited. Shatil-Cohen *et al.* (2011) have also shown that either drought or exogenous application of ABA triggered a reduction of the permeability coefficient (and thus *K*_leaf_) of bundle sheath cells, but not that of the mesophyll cells. As previously mentioned, this suggests a partial hydraulic isolation of mesophyll cells from the xylem–bundle sheath–stomata pathway, thereby possibly delaying ABA delivery. Since certain aquaporins have been reported to have a CO_2_ channel role (Otto *et al.*, 2010; Uehlein *et al.*, 2012), it may be that *g*_m_ is less sensitive to drought stress than *g*_sw_ or *K*_leaf_ because regulation of mesophyll aquaporins is expected to take place at a more advanced drought stress due to hydraulic compartmentalization.

### Delayed *g*_m_ response to drought favours water use efficiency in hybrid poplar

One aim of the present study was to compare two clones of contrasting drought tolerance in order to determine if they differ in their *g*_sw_, *K*_leaf_, and *g*_m_ relationships. Both drought-tolerant Okanese and drought-sensitive Assiniboine exhibited a similar *g*_m_ threshold response to drought (Fig. 3), potentially suggesting a partial hydraulic isolation of the mesophyll cells. This in turn allowed a delay in the decline of *A*, thereby favouring a higher water use efficiency (WUE) during the early stages of drought. However, those clones differed in their vulnerability to stem cavitation and in the Ψ_soil_ at which their respective minimal *g*_sw_ was reached (Fig. 4), as observed in other hybrid poplar studies (Silim *et al.*, 2009; Arango-Velez *et al.*, 2011; Larcheveque *et al.*, 2011). Although the vulnerability curves in the present study showed that Okanese allowed more cavitation to occur at similar Ψ_stem_ values than Assiniboine (Fig. 6), both clones modulated *g*_sw_ to maintain Ψ_stem_ at levels preventing conductivity to drop below ~25%, a feature previously observed for those same clones under severe drought stress (Arango-Velez *et al.*, 2011). While Okanese maintained similar *A*_Ca=380_ and *g*_m_ to Assiniboine at a given *g*_sw_, it adopted a more conservative water use strategy by lowering *g*_sw_ more than Assiniboine under increasing soil water depletion (see also Arango-Velez *et al.*, 2011). Hence, Okanese exhibited a higher intrinsic water use efficiency (WUE_i_) than Assiniboine at comparable Ψ_soil_ (it also displayed a higher *g*_m_/*g*_sw_ ratio; data not shown). Such maintenance of high *g*_m_/*g*_sw_ ratios has recently been identified as an important trait to improve WUE in cultivated plants (Barbour *et al.*, 2010; Flexas *et al.*, 2013).

### Conclusion

When subjecting young hybrid poplar clones of contrasting drought tolerance to a short period of water stress, *K*_leaf_ and *g*_sw_ decreased monotonically in concert, while *g*_m_ remained constant over most of its range, causing a delay in the decline of *A*. Only when *g*_sw_ fell below an approximate threshold of 0.15mol m^–2^ s^–1^, ~33% (Assiniboine) to 40% (Okanese) of maximum *g*_sw_, was a decrease in *g*_m_ observed (and this even when measured at a normalized *C*_i_ to remove the photorespiratory bias modelled by Tholen *et al.*, 2012), in accordance with the recent results of Ferrio *et al.* (2012). The present results thus support the recent suggestion that the bulk mesophyll is partially isolated (i.e. buffered) from the major transpiration pathway delivering ABA to the stomata (Shatil-Cohen *et al.*, 2011; Pantin *et al.*, 2013). The delayed photosynthetic decline mediated by such a *g*_m_ threshold response resulted in enhancing WUE in the early stage of drought.

## Supplementary data

Supplementary data are available at *JXB* online.

Figure S1. Apparent sensitivity of mesophyll conductance (*g*_m_) to intercellular CO_2_ mole fraction (*C*_i_): comparison of the responses observed under three different *g*_m_ estimation methods.

Figure S2. Schematic of the large plant cuvette used in the low *C*_a_ experiment.

Figure S3. Continuous measurements of photosynthesis (*A*), mesophyll conductance (*g*_m_), stomatal conductance to water (*g*_sw_), and leaf hydraulic conductance (*K*_leaf_) during 5 d of water depletion.

Figure S4. Relationship between leaf hydraulic conductance (*K*_leaf_) and leaf water potential (Ψ_leaf_) during 5 d of water depletion.

Supplementary Data
