# Supplementary material for: Threshold response of mesophyll CO2 conductance to leaf hydraulics in highly transpiring hybrid poplar clones exposed to soil drying
Source: J Exp Bot. 2013 Dec 24;65(2):741–53. doi: 10.1093/jxb/ert436 (PMC3904724; doi:10.1093/jxb/ert436)
Supplement: Supplementary Data [file supp_65_2_741__index.html]

Threshold response of mesophyll CO2 conductance to leaf hydraulics in highly transpiring hybrid poplar clones exposed to soil drying — Supplementary Data 

# Threshold response of mesophyll CO2 conductance to leaf hydraulics in highly transpiring hybrid poplar clones exposed to soil drying

## Supplementary Data

Data files

**Files in this Data Supplement:**

- Supplementary Data - Supplementary Data
