# Supplementary material for: Threshold response of mesophyll CO2 conductance to leaf hydraulics in highly transpiring hybrid poplar clones exposed to soil drying
Source: J Exp Bot. 2013 Dec 24;65(2):741–53. doi: 10.1093/jxb/ert436 (PMC3904724; doi:10.1093/jxb/ert436)
Supplement: Supplementary Data [file supp_ert436_jexbot107490_file001.pdf]

Journal of Experimental Botany, Threshold response of mesophyll CO_2 conductance to leaf hydraulics in highly transpiring hybrid poplar clones exposed to soil drying

G. Théroux Rancourt, G. Éthier, S. Pepin

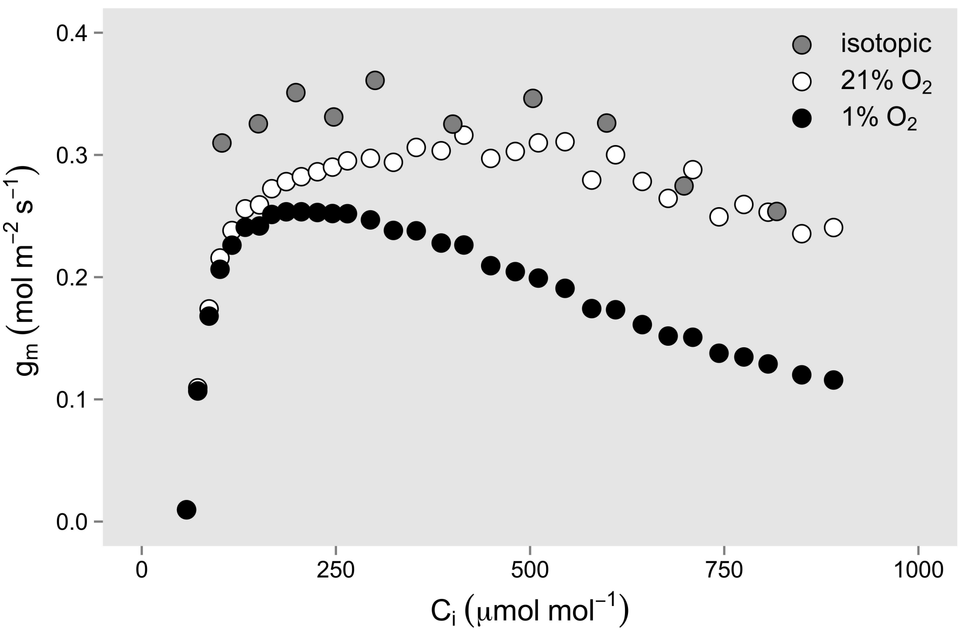

Supplemental Figure S1. Apparent sensitivity of mesophyll conductance (g_m) to intercellular CO_2 mole fraction (C_i) – comparison of the responses observed under three different g_m estimation methods (see Tholen et al. (2012) for details on the isotopic method).

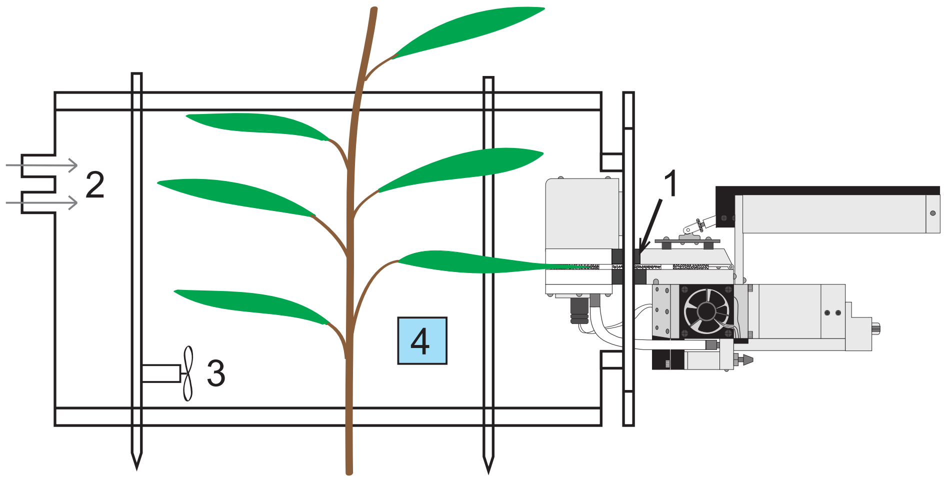

Supplemental Figure S2. Schematic of the large plant cuvette used in the low C_a experiment.

A Li-Cor LI-6400XT equipped with a LCF head was inserted inside a cylindrical cuvette (0.3 m diameter and 0.2 m long; polypropylene walls and acrylic top and bottom plates) through an opening. A brass spacer was added between the LCF and IRGA head and sealed with neoprene gasket (1). Air was pumped inside the plant cuvette through holes on the upper part (2) and was mixed inside with two 12 V DC fans (3). A second sealed opening was used to facilitate the insertion of a leaf inside the LI-6400XT chamber using tweezers (4).

LI-6400XT image courtesy of LICOR Biosciences and used under permission.

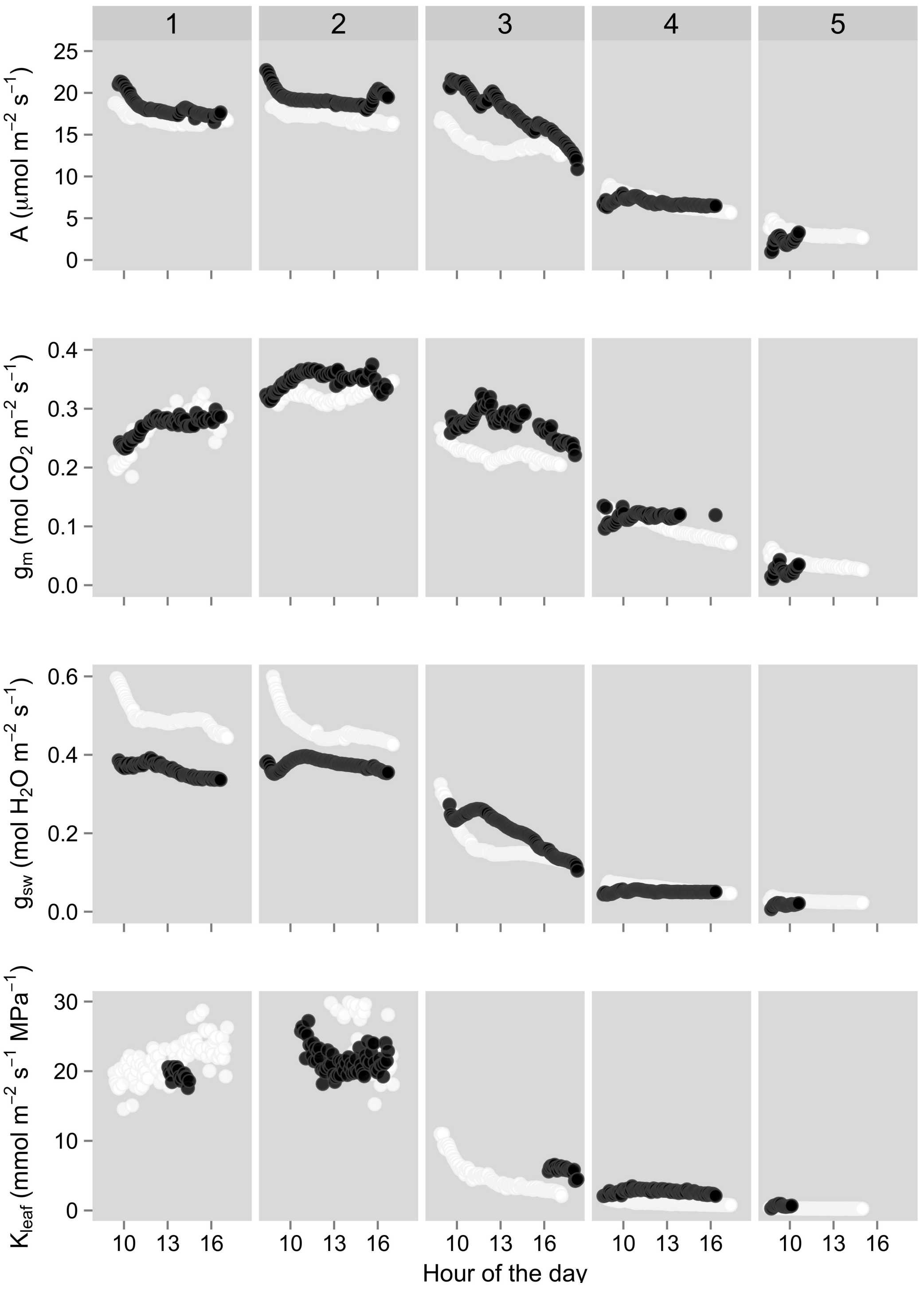

Supplemental Figure S3. Continuous measurements of photosynthesis (A), mesophyll conductance (g_m), stomatal conductance (g_{sw}), and leaf hydraulic conductance (K_{leaf}) during five days of water depletion. One plant of each clone was monitored (drought tolerant Okanese: black dots; drought sensitive Assiniboine: white dots). Missing data in K_{leaf} for Okanese on day 1 and 3 are due to technical problems with data acquisition and leaf psychrometers.

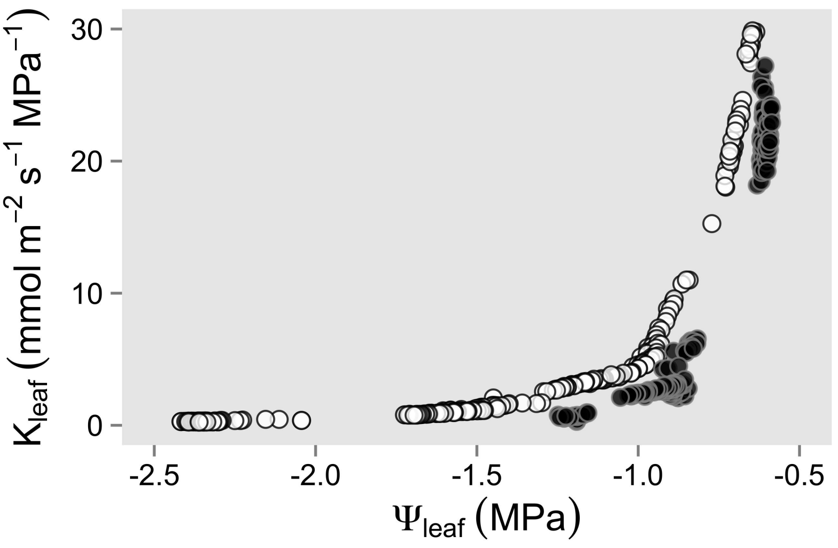

Supplemental Figure S4. Relationship between leaf hydraulic conductivity (K_{leaf}) and leaf water potential (Ψ_{leaf}) during five days of water depletion.
