# Supplementary figures and images for: Threshold response of mesophyll CO2 conductance to leaf hydraulics in highly transpiring hybrid poplar clones exposed to soil drying
Source: J Exp Bot. 2013 Dec 24;65(2):741–53. doi: 10.1093/jxb/ert436 (PMC3904724; doi:10.1093/jxb/ert436)

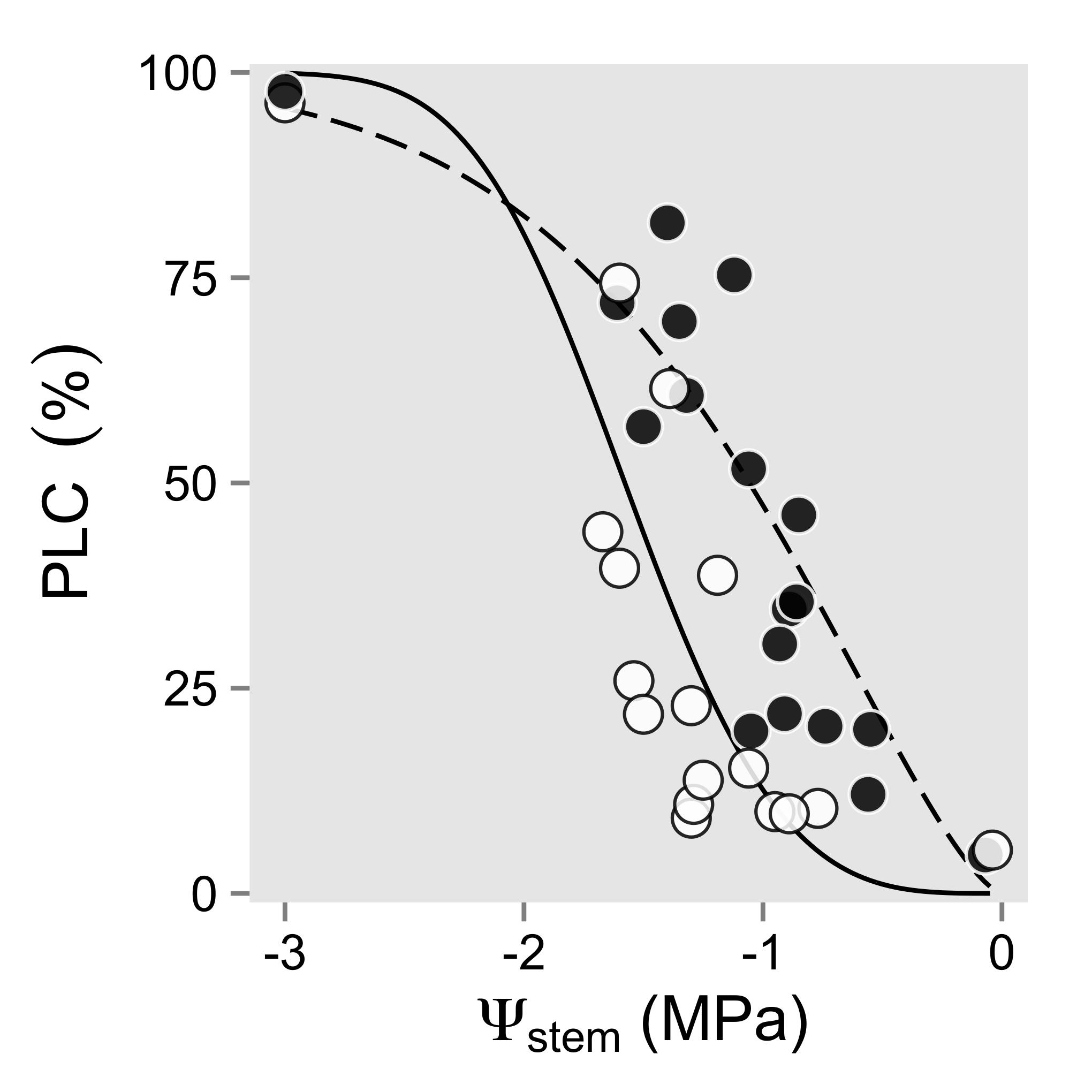

Supplement: Supplementary Data [file supp_ert436_jexbot107490_file002.tif]
